# Revisiting the optical bandgap of semiconductors and the proposal of a unified methodology to its determination

**DOI:** 10.1038/s41598-019-47670-y

**Published:** 2019-08-02

**Authors:** A. R. Zanatta

**Affiliations:** Instituto de Física de São Carlos, USP, São Carlos, 13560-970 SP Brazil

**Keywords:** Semiconductors, Semiconductors

## Abstract

Along the last two centuries, the story of semiconductor materials ranged from a mix of disbelief and frustration to one of the most successful technological achievements ever seen. Such a progress comprised the development of materials and models that, allied to the knowledge provided by spectroscopic techniques, resulted in the (nowadays) omnipresent electronic gadgets. Within this context, optically-based methods were of special importance since, amongst others, they presented details about the electronic states and energy bandgap E_gap_ of semiconductors which, ultimately, decided about their application in devices. Stimulated by these aspects, this work investigated the semiconductors silicon, germanium, and gallium-arsenide in the crystalline (bulk and powder) and amorphous (film) forms. The detailed analysis of the experimental results indicates that accurate E_gap_ values can be obtained by fitting a sigmoid (Boltzmann) function to their corresponding optical absorption spectra. The method is straightforward and, contrary to the traditional approaches to determine E_gap_, it is exempt from errors due to experimental spectra acquisition and data processing. Additionally, it complies with the requirements of direct, indirect, and amorphous bandgap semiconductors, and it is able to probe the (dis)order of the material as well. In view of these characteristics, a new−unified methodology based on the fitting of the absorption spectrum with a Boltzmann function is being proposed to efficiently determine the optical bandgap of semiconductor materials.

## Introduction

### Semiconductors and optical spectroscopy

For a long time, the distinctive electrical behavior exhibited by semiconductor materials has fascinated the humankind^1,2^. Since the very first studies by Alessandro Volta of the so-called *cattivi conduttori* in the 18th century^3^; passing by all the experimental work of Humphry Davy^4^, Michael Faraday^5^ and Wilhelm Hittorff^6^; and the discovery of the photovoltaic^7,8^ and rectification effects^9,10^; it was a long way until some of the properties of the semiconductors have been (partially) elucidated by the quantum theory of electrons developed by Alan Wilson in 1931 ^11,12^. Such a long journey took place because of the suitability of the vacuum electron tubes in electronic applications^13,14^ but, mainly, because of the absence of good-quality (*i.e*., impurity-controlled) semiconductor materials − rendering erratic or non-reproducible series of experimental results. In fact, the skepticism involving these materials was so intense that the word semiconductor (*halbleiter* in German) − suggesting its real electrical characteristics − was introduced only in 1911 by Josef Weiss, at that time, a student of Professor Johann Koenigsberger^15,16^. Even though the theoretical basis provided by Alan Wilson represented a significant leap forward in the semiconductors science and technology, it was the emergence of both silicon (Si) and germanium (Ge), as semiconductor materials, that effectively prompted the extraordinary achievements in the field − initially, with the development of p-n junctions^17–23^ and, later on, with the realization of semiconductor triodes or, the transistors^24–26^. Further technological progress included: solar cells^27,28^, integrated circuits^29,30^, light-emitting diodes^31^, solid-state laser sources^32^, charge-coupled devices^33^ etc. − as well as the emergence of new (manmade) materials like gallium-arsenide (GaAs)^34,35^, for example.

In retrospect, it is clear the role played by new−improved semiconductor materials and theoretical models in the (ubiquitous) modern industry of electronic devices. The advent of novel experimental characterization techniques was also decisive in the whole process, either providing more sensitive or different physical−chemical information, and it is about one of these techniques that this work is related to.

Roughly, the optical properties of any material are determined by its interaction with an electromagnetic radiation field and comprises transmission, absorption, emission, reflection, refraction, diffraction, or scattering effects. In the specific case of semiconductors, these optical properties (mostly in the ultraviolet, visible, or infrared ranges) are related to the characteristics of the electronic bands of semiconductors or, ultimately, to their atomic structure, particular atoms, and chemical bonding^36–38^. In fact, the first reports involving Si, Ge, and GaAs came out in the 1950–1960’s^39–45^, and they were important not only for presenting the optical properties of these materials but, specially, by expanding our concepts in modern solid-state physics. Studies of the optical absorption edges of semiconductor materials give information about the states nearby the valence and conduction bands as well as their corresponding energy separation (or forbidden energy bandgap E_gap_)^46^ − both parameters essential to decide the semiconductor abilities regarding future device application. Apart from differences concerning the results presented by unlike experimental methods^47–49^, the bandgap of any semiconductor can be obtained from electrical conductivity^50^, Hall effect^51^, photoconductivity^52^, or optical absorption measurements. Whereas some of these methods are temperature-dependent (electrical conductivity and Hall effect) and/or surface-sensitive (photoconductivity), those ones involving optical processes are rather simple. Besides, optical spectroscopic methods have many unique and attractive features, such as^53^: (a) they are non-destructive, contactless, and requires minimum (or no) sample preparation; (b) depending on the instrumentation involved, they are fast (≤10^−6^ sec) and consistent with either high spatial resolution (~10^−7^ m) or mapping−imaging experiments; (c) they are able to provide atomic−structural information and, hence, support or complement elemental analyses − in special those ones related to the presence of impurities and/or defects; and (d) along with some of the previous attributes, more recently, they are available in the form of portable (low-consumption) systems alloying their use in the most diverse circumstances (high-temperature and/or hazardous environments, *in-situ* real-time experiments, *in-line* industrial processing etc.). Spectroscopic ellipsometry can also present the optical properties of semiconductor or dielectric materials although, in certain cases, the information is limited by aspects involving theoretical models, sample surface quality, tabulated data (in the case of thin films), and light penetration depths, for example^54^.

### Optical absorption and optical bandgap

At present, most of our knowledge about the optical absorption of crystalline semiconductors derive from models in which the electrons receive a quantum mechanics approach and the photons are described by classic electromagnetic waves. Within this semi-classical framework, the optical absorption coefficient α, at the photon energy E ($=\hslash {\rm{\omega }}$), of an electron being excited from the valence (VB) to the conduction (CB) band, is subjected to the transition rate:

1 $${{\rm{W}}}_{{\rm{VB}}\to {\rm{CB}}}=\frac{2\pi }{\hslash }{|{\rm{M}}|}^{2}{\rm{g}}({\rm{E}}),$$

where M and g(E) stand for the (coupling) transition matrix element and the (joint electron−hole) density of states. Taking into account transitions with negligible (or no) changes in the electron wave-vector $\overrightarrow{{\rm{k}}}$ ($\overrightarrow{{{\rm{k}}}_{{\rm{f}}}}\approx \overrightarrow{{{\rm{k}}}_{{\rm{i}}}}$) – in which case the optical transitions are called direct or vertical – along with aspects regarding atom bonding and selection rules^38^, the absorption coefficient α(E) of a semiconductor is expected to behave like:

$${{\rm{\alpha }}}_{{\rm{dir}}}({\rm{E}} < {{\rm{E}}}_{{\rm{gap}}})=0,$$

and

2 $${{\rm{\alpha }}}_{{\rm{dir}}}({\rm{E}}\ge {{\rm{E}}}_{{\rm{gap}}})\propto {({\rm{E}}-{{\rm{E}}}_{{\rm{gap}}})}^{1/2}.$$

In such a case, the semiconductor is said to exhibit optical direct bandgap and its E_gap_ value can be determined by extrapolating the linear least squares fit of α^2^ to zero, in a “α^2^ *versus* E” plot. The method is simple and very convenient in determining the E_gap_ of direct bandgap semiconductors − in spite of some inadequacies involving the semi-classical model like^38^: (a) no Coulomb attraction has been considered which, notably at lower temperatures, enhances α_dir_(E); (b) the fact that extrinsic absorption (due to defect states and impurities) may originate α_dir_(E < E_gap_) ≠ 0; and (c) that the parabolic-like α_dir_(E ≥ E_gap_)∝(E − E_gap_)^1/2^ shape is valid only at $\overrightarrow{{\rm{k}}}\approx 0$, that could not be valid in certain band structures and, definitely, is not applicable when E ≫ E_gap_.

Contrary to the previous case, indirect (or non-vertical) optical transitions involve a photon and (at least) one phonon in order to comply with the conservation of momentum. As a consequence, the transition rates W_VB→CB_ taking place in indirect optical bandgap semiconductors are smaller than those typically verified in the direct bandgap ones, and the corresponding optical absorption coefficient α(E) can be written as (see ref.^37^ for a complete description):

$${{\rm{\alpha }}}_{{\rm{ind}}}({\rm{E}} < {{\rm{E}}}_{{\rm{gap}}})=0,$$

and

3 $${{\rm{\alpha }}}_{{\rm{ind}}}({\rm{E}}\ge {{\rm{E}}}_{{\rm{gap}}})\propto {({\rm{E}}\pm \hslash {\rm{\Omega }}-{{\rm{E}}}_{{\rm{gap}}})}^{2},$$

where $\hslash {\rm{\Omega }}$ denotes the energy of a phonon being emitted ($+\hslash {\rm{\Omega }}$) or absorbed ($-\hslash {\rm{\Omega }}$). In most of the situations, the contribution owing to $\hslash {\rm{\Omega }}$ can be disregarded and, analogously to the case of direct bandgap semiconductors, the indirect E_gap_ values can be obtained from the extrapolation of the linear least squares fit of α^1/2^ to zero (“α^1/2^ *versus* E” plot).

Put side by side, the first-order (direct) and phonon-assisted (indirect) nature of the optical processes, typical of crystalline semiconductors, give rise to steeper α_dir_(E) profiles and to the presence of absorption tails (nearby E_gap_) due to higher frequency (or multi-) phonon absorption in α_ind_(E). Furthermore, both α_dir_(E) and α_ind_(E) – and corresponding E_gap_ values − are affected by the local temperature^55^, the presence of external strong electric^56^ or magnetic^57^ fields, and the physical−chemical characteristics (including doping−alloying effects) of the semiconductor material as well^36^.

In fact, when structural–electronic disorder applies, optical absorption transitions require a different approach and a number of procedures are in use to determine the E_gap_ of amorphous (or non-crystalline) semiconductors. Strictly, because of the presence of tail states nearby the valence and conduction bands, the E_gap_ of amorphous semiconductors is defined by the extrapolation of the joint density of states^58–60^. This can be achieved by imposing certain restrictions to the optical absorption processes as, for example: (a) absence of $\overrightarrow{{\rm{k}}}$-conservation; (b) a constant momentum transition matrix element (as expected for phonon-assisted transitions); and (c) similar to the crystalline case, the density of electron states close to the VB and CB extrema is proportional to the square root of the photon energy. Such approach was originally proposed by Tauc *et al*.^61^, from which the E_gap_ (or, more appropriately, the Tauc’s bandgap E_Tauc_) of any amorphous semiconductor can be determined by extrapolating the linear least squares fit of (α · E)^1/2^ to zero [“(α · E)^1/2^ *versus* E” plot]. Another possibility, assuming a constant dipole transition matrix element, was adopted by Cody *et al*.^62^, in which the Cody’s bandgap E_Cody_ arises from the extrapolation of the linear least squares fit of (α/E)^1/2^ to zero [“(α/E)^1/2^ *versus* E” plot]. Rigorously, neither the transition matrix elements are absolutely constant, nor the VB and CB band edges are perfectly parabolic-shaped, but these effects cancel each other out so that the methods provide reasonable estimates of E_gap_^59^. Most probably because E_Tauc_ > E_Cody_, the Tauc’s method is the preferred one to evaluate the E_gap_ of amorphous (or glassy) materials and, despite its clear purpose, it has been routinely (and incorrectly) applied to study crystalline and/or highly-doped semiconductors^63^. Instead, the bandgap of amorphous semiconductors can be defined by taking the photon energy at which the optical absorption coefficient reaches 10^3^ or 10^4^ cm^−1^, rendering the so-called isoabsorption E_03_ or E_04_ bandgaps^59,64^. Notwithstanding its convenience, this procedure is useful only when α(E) ≥ 10^3^ cm^−1^ − as typically verified in samples with thicknesses in the (sub-)micrometer range^65^.

Regardless of the method chosen to determine the optical bandgaps of either crystalline or amorphous semiconductors, all of them are influenced by the α(E) spectrum and its posterior data analysis. In the former, the α(E) spectrum is susceptible to experimental aspects (measurement details, sample thickness etc.) and, most importantly, to the mathematical expression chosen to its calculation. In the latter, as long as the correct graphical representation has been taken, E_gap_ values can differ by many meV simply by selecting a different range to perform the linear regression analysis.

Motivated by these issues, the present work investigated the optical spectra of Si, Ge and GaAs samples − under the crystalline (bulk and powder) and amorphous (film) forms. Based on these investigations, the work proposes an alternative methodology to determine the E_gap_ of semiconductor materials, from their experimental transmittance and/or reflectance spectra, which is free from problems regarding the measurement−calculation of α(E) and corresponding data analysis.

## Experimental Results and Discussion

### Optical measurements & The α(E) coefficient

Optical transmission (*reflection*) is used to describe the process by which a fraction of an incident electromagnetic field: leaves a surface or medium from a side other than the incident one (*returns into the same hemisphere whose base is the surface and that contains the incident one*). Hence, the following nomenclature applies^66,67^: spectral transmittance (or transmission coefficient) T = l_T_/l_To_ (ratio of the transmitted l_T_ to the incident l_To_ radiation flux), and spectral reflectance (or reflection coefficient) R = l_R_/l_Ro_ (ratio of the reflected l_R_ to the incident l_Ro_ radiation flux) − as determined from their geometries and appropriate specular or diffuse reflection standards. Correspondingly, the optical absorption coefficient (or absorptance) α is defined as the fraction of the incident radiation flux that was converted into neither transmission nor reflection fluxes. Besides, α is known to change with the photon energy E (just like T and R) and to exhibit a logarithmic dependence with the optical path length d, giving rise to the so-called Bouguer-Lambert-Beer absorption law^68–70^ – henceforth, our α_BLB_(E):

4 $${{\rm{\alpha }}}_{{\rm{BLB}}}({\rm{E}})=+\,\frac{{\rm{1}}}{{\rm{d}}}\,\mathrm{ln}(\frac{{\rm{1}}}{{\rm{T}}}).$$

whereas Eq. (4) is the simplest one can conceive to represent the optical absorption taking place in semiconductor materials, further improvements should include the reflectance R. In this case, the radiation across a (d thick, polished, uniform) semiconductor slab can be described by the following flux sequence^65^: incoming l_To_; (1 − R)l_To_ traversing the first interface; (1 − R)l_To_ exp(−αd) reaching the second interface; and (1 − R)^2^ l_To_ exp(−αd) leaving the slab − corresponding exactly to the transmitted radiation flux l_T_ when the product αd is large. Such “simple”, or simplified, optical absorption coefficient can be expressed by − our α_simp_(E):

5 $${{\rm{\alpha }}}_{{\rm{simp}}}({\rm{E}})=+\,\frac{{\rm{1}}}{{\rm{d}}}\,\mathrm{ln}[\frac{{(1-{\rm{R}})}^{{\rm{2}}}}{{\rm{T}}}].$$

If, on the contrary, multiple internal reflections are considered (*i.e*., αd is small) the “complete” optical absorption coefficient – our α_comp_(E) – will be given by^65,71^:

6 $${{\rm{\alpha }}}_{{\rm{comp}}}({\rm{E}})=+\,\frac{{\rm{1}}}{{\rm{d}}}\,\mathrm{ln}[\frac{{({\rm{1}}-{\rm{R}})}^{{\rm{2}}}}{{\rm{2T}}}+\sqrt{\frac{{({\rm{1}}-{\rm{R}})}^{{\rm{4}}}}{{{\rm{4T}}}^{{\rm{2}}}}+{{\rm{R}}}^{{\rm{2}}}}].$$

Therefore, for the purposes of the present work, the optical absorption coefficients were arranged according to three classes: α_BLB_(E), α_simp_(E), and α_comp_(E) – as obtained from the very same T, R, and d experimental data.

Since the powdered samples exhibit only diffuse reflection, their α(E) values were calculated from a different approach. In such case, a basic two-flux radiation model – taking into account the incoming and the diffusively reflected light^72^ – yields a function F(R_∞_) that relates the apparent absorption K and diffuse reflection S coefficients via the Schuster-Kubelka-Munk formula^73–76^:

7 $${\rm{F}}({{\rm{R}}}_{\infty })=\frac{{(1-{{\rm{R}}}_{\infty })}^{2}}{2{{\rm{R}}}_{\infty }}=\frac{{\rm{K}}}{{\rm{S}}},$$

where R_∞_ is the diffuse reflectance of the sample – as referred to a non-absorbing standard. The validity of F(R_∞_) relies upon the condition that the sample is thick, densely-packed, and constituted by randomly-shaped particles whose sizes are comparable to (or smaller than) the wavelength of the incoming radiation. It is also expected that, within the measured range: the sample does not emit light, and that the diffuse reflection coefficient S does not change appreciably^77^. Despite the fact that most of these criteria comply with the present samples characteristics and measurements conditions, rigorously, the uncertainty regarding S makes F(R_∞_) a pseudo-absorption function. Nonetheless, the F(R_∞_) ~ α_SKM_(E) approximation is good enough (and widely-accepted^78,79^ to calculate the optical bandgap of semiconductors from their diffuse reflectance spectra.

Equations (4) to (7) form the basis of the following discussion in which the optical bandgaps of Ge, Si, and GaAs were determined from their experimental transmittance and/or reflectance spectra. The discussion compares the E_gap_ values obtained by the traditional approaches (*i.e*., linear regression analysis of α^2^, α^1/2^, and Tauc methods) with those provided by the Boltzmann function that fits the experimental α(E)– as is being proposed here for the first time. For completeness, the discussion also includes the E_gap_’s evaluated from less common procedures such as, for example: the plain extrapolation of the transmittance or reflectance spectra^80,81^; the energy (or wavelength) derivative of the transmittance or reflectance spectra^82,83^; the extrapolation of the optical absorption coefficient α(E)^79,84,85^ etc. In addition to the proposal of a new–unified methodology, this work aims to show the main (conceptual and numerical) differences between the various approaches to estimate the E_gap_ of semiconductor materials. In particular, it wants to stress that the Tauc bandgap refers to amorphous^58,59,61,86^, nano-structured^87^, and/or (mixed-phase^88^) poly-crystalline materials^58,63^, and is described, exclusively, by and its distinctive “(α · E)^1/2^ *versus* E” plot. Hence, to associate the Tauc bandgap or Tauc plot with anything different from the previous cases is misleading^89–91^ and should be avoided.

### Crystalline (wafer) Ge, Si, and GaAs

**Figure 1:**
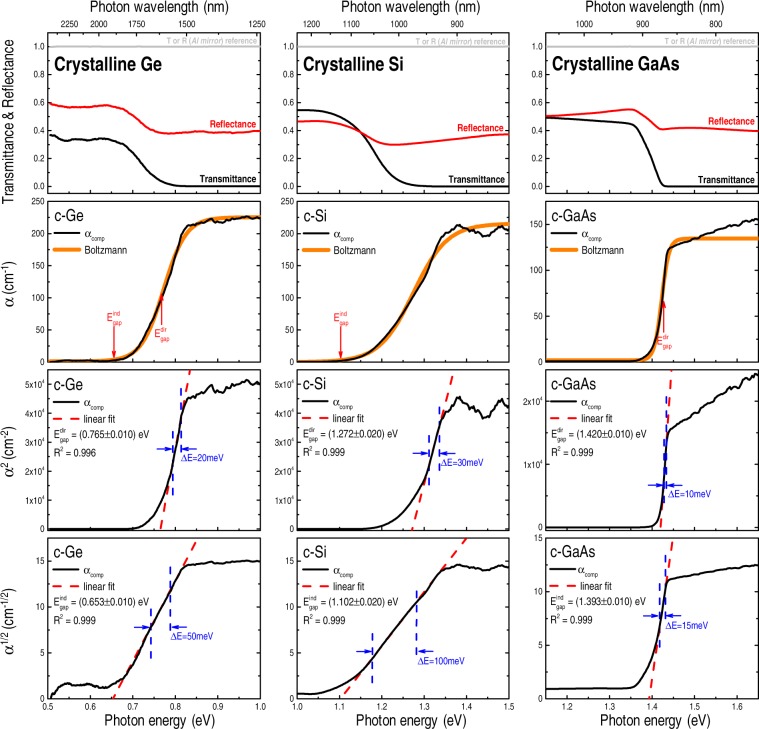
Optical transmittance, reflectance, and absorption coefficient α(E) [after Eq. (6)] spectra of crystalline Ge, Si, and GaAs samples. The measurements were carried out at room-temperature and were corrected by system response and specular reflection (aluminum mirror) standard. The optical bandgaps, both direct and indirect, were determined from their corresponding α^2^ and α^1/2^ graphic representations – clearly indicating the resultant linear fit, fitting range ΔE, and R^2^ *goodness-of-fit* measure. The errors in the E_gap_ values refer to data dispersion due to different measurements runs. The α *versus* E graphs also show the Boltzmann functions used to fit α(E), and the E_gap_ values (as obtained from their respective linear regression analysis).

The optical transmittance (T), reflectance (R), and absorption coefficient (α_comp_) spectra of the crystalline Ge, Si, and GaAs (wafer) samples are shown in Fig. 1. Their corresponding α^2^ and α^1/2^ *versus* photon energy E plots are presented as well. In this case, the figure also indicates the E_gap_ values – as obtained from the linear least squares fit procedure (clearly indicating the typical fitting range ΔE and the R^2^ predictive power, or *goodness-of-fit* measure).

At first glance, Fig. 1 contains two remarkable aspects: (1) the rather limited energy ranges (ΔE ≤ 100 meV) providing good linear fits, and (2) that, despite the origin of the optical transitions, both direct and indirect E_gap_ values can be achieved from the respective α^2^ and α^1/2^ *versus* E plots. Whereas the reduced energy ranges are related to the optical processes taking place in a crystalline semiconductor (*i.e*., first-order *versus* phonon-assisted – at very different transition rates), it is clear that, *per se*, the α^2^ and α^1/2^ *versus* E plots cannot be used to decide on the real nature of the optical bandgaps^36–38^ – in the present case: direct (GaAs), indirect (Si), and both direct and indirect (Ge). Moreover, in the absence of specific rules, the whole process (including graphical representation, fitting range, and *goodness-of-fit* target) is highly susceptible to the operator’s intervention. In the present work one considered in the fitting procedure: all possible graphical representations; a minimum R^2^ of 0.99; and, whenever applicable, the energy region corresponding to the optical absorption edge (but beyond the tail states) of the plots.

Additionally, Fig. 1 shows the fitting of the α_comp_(E) spectra according to the sigmoid-Boltzmann function:

8 $${\rm{\alpha }}({\rm{E}})={{\rm{\alpha }}}_{{\rm{\max }}}+\frac{{{\rm{\alpha }}}_{{\rm{\min }}}-{{\rm{\alpha }}}_{{\rm{\max }}}}{{\rm{1}}+\exp (\frac{{\rm{E}}-{{\rm{E}}}_{0}^{{\rm{Boltz}}}}{{\rm{\delta }}E})},$$

where α_min_ (α_max_) stands for the minimum (maximum) absorption coefficient; ${{\rm{E}}}_{{\rm{0}}}^{{\rm{Boltz}}}$ is the energy coordinate at which the absorption coefficient is halfway between α_min_ and α_max_; and δE is associated with the slope of the sigmoid curve^92,93^. Also, contrary to the analysis of the α^2^ and α^1/2^ *versus* E plots, the fitting of the Boltzmann functions comprised a ~500 meV energy range centered in between the min-max α_comp_(E) values.

Taking into consideration the typical profile of the α(E) curves (S-shaped and constrained by a pair of horizontal asymptotes as E → ±∞), their association with a sigmoid-Boltzmann function [Eq. (8)] was quite predictable. Most of all, the Boltzmann function is simple and (as will be shown) consistent with the optical processes regarding the experimental determination of E_gap_. However, whereas R^2^ is a significant (and well-established) *goodness-of-fit* parameter in linear regression analysis, there are many different ways, and no absolute consensus, to calculate the (pseudo-)R^2^ of nonlinear functions as, for example, the sigmoid-Boltzmann one^94–96^. Therefore, for the purposes of the present work one accepted that the Boltzmann functions provided good fits as long as: they reproduced a high portion of the α(E) spectrum, and they presented the variables α_min,max_, ${{\rm{E}}}_{{\rm{0}}}^{{\rm{Boltz}}}$, and δE with little uncertainty. Moreover, despite some deviations at high photon energies (particularly with the GaAs sample in Fig. 1), the Boltzmann functions were very effective to reproduce the α(E) regions corresponding to the optical absorption edges – exactly the ones responsible to define E_gap_.

**Figure 2:**
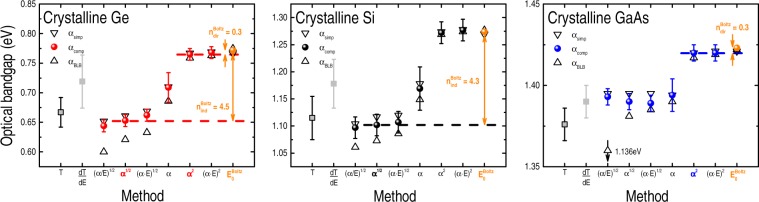
Optical bandgap E_gap_ values of crystalline Ge, Si, and GaAs – as determined from different experimental methods: extrapolation (*energy derivative*) of the transmittance curve T (*dT/dE*); Cody’s bandgap [(α/E)^1/2^ *versus* E plot]; indirect E_gap_ [α^1/2^]; Tauc’s bandgap [(α · E)^1/2^]; extrapolation of α(E); direct E_gap_ [α^2^]; inappropriate (but extensively applied^89–91^) version of the Tauc’s bandgap [(α · E)^2^]; and after fitting the α(E) spectra with Boltzmann functions (${{\rm{E}}}_{{\rm{0}}}^{{\rm{Boltz}}}$). The figures include data from α_BLB_(E) [Eq. (4)], α_simp_(E) [Eq. (5)], and α_comp_(E) [Eq. (6)]. Error bars correspond to data dispersion due to different sets of measurements–analyses and, for clarity reasons, were indicated only in the T-, dT/dE-, and α_comp_-related bandgap values. In all cases ${{\rm{E}}}_{{\rm{0}}}^{{\rm{Boltz}}}\pm 0.005$ eV, *i.e*., on the order of (or below) the typical spectrum resolution of ~10 nm.

The information contained in Fig. 1 is complemented by the data of Fig. 2 that displays the E_gap_ of crystalline Ge, Si, and GaAs – as obtained from different methods and α(E) expressions. The corresponding experimental error due to various measurements runs and data processing is also presented, clearly denoting the failure of certain methods: either because of higher uncertainty [plain extrapolation of T and α(E)] and/or because of larger noise (dT/dE). The expected direct and indirect optical bandgaps – following the traditional α^2^ and α^1/2^ *versus* E plots – were also indicated (by horizontal dashed lines) and compared with the results obtained from the Boltzmann fittings (${{\rm{E}}}_{{\rm{0}}}^{{\rm{Boltz}}}$). In addition to the different E_gap_ values shown in Fig. 2, it is worth noticing that, within the experimental error: (a) the Cody’s, indirect, and Tauc’s optical bandgaps are almost the same; (b) systematically, the α_BLB_(E) data yields underestimated E_gap_’s; and (c) the ${{\rm{E}}}_{{\rm{0}}}^{{\rm{Boltz}}}$ values provided by the α_BLB_(E), α_simp_(E), and α_comp_(E) spectra are identical. Whereas items (a) and (b) are expected due to the second-order (phonon-assisted) nature of the optical transitions and because of the omission of the light reflection contributions, respectively, the insensitivity of ${{\rm{E}}}_{{\rm{0}}}^{{\rm{Boltz}}}$ to the various α(E) formulas emphasizes its suitability in determining the optical bandgaps of crystalline Ge, Si, and GaAs.

**Figure 3:**
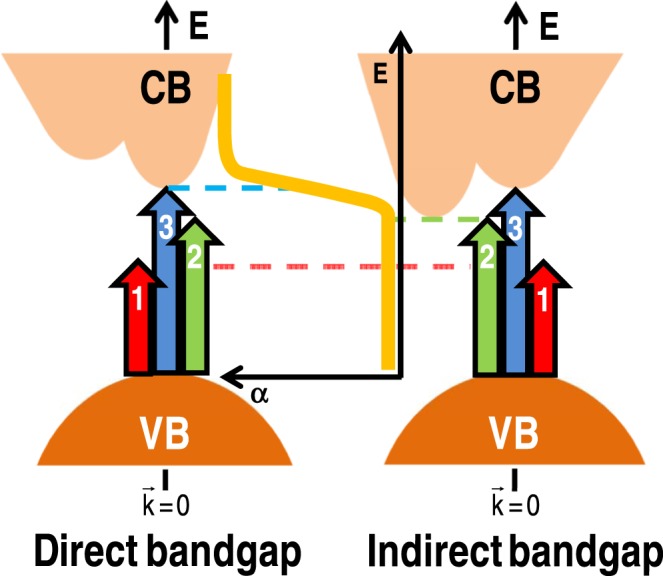
Simplified energy diagram illustrating the valence (VB) and conduction (CB) bands of direct and indirect bandgap semiconductors. The sketch of a typical absorption coefficient spectrum (α *versus* E) and some optical transitions (denoted by 1, 2, and 3) are represented as well. According to the figure, as the photon energy E increases, the following optical phenomena take place: 1– below bandgap (no absorption); 2– absorption onset (due to defects and/or phonon-assisted processes); and 3– high absorption edge (maximum of the optical absorption rate).

Indeed, the ensemble of experimental results suggests that there exists a central energy ${{\rm{E}}}_{{\rm{0}}}^{{\rm{Boltz}}}$ (and its corresponding distribution δE owing to differences in the nature of the bandgaps, presence of disorder etc.) around which most of the optical transitions take place. According to this picture, in which the Boltzmann function reproduces the α(E) spectrum, the E_gap_’s of crystalline Ge, Si, and GaAs can be defined by means of the following empirical relationship:

9 $${{\rm{E}}}_{{\rm{gap}}}^{{\rm{Boltz}}}={{\rm{E}}}_{{\rm{0}}}^{{\rm{Boltz}}}-{{\rm{n}}}_{\mathrm{dir}-\mathrm{ind}}^{{\rm{Boltz}}}\times {\rm{\delta }}E,$$

with ${{\rm{n}}}_{{\rm{dir}}}^{{\rm{Boltz}}}\sim 0.3$ and ${{\rm{n}}}_{{\rm{ind}}}^{{\rm{Boltz}}} \sim {\rm{4.3}}$, as determined from the corresponding direct and indirect experimentally determined optical bandgaps of Figs 1 and 2 (also, see SuppInfo_Part 1). Compared with the values obtained from the traditional α^2^ and α^1/2^ methods, Eq. (9) is able to provide E_gap_’s with an accuracy (absolute error) below 10 meV. The rather good association between the optical E_gap_’s, as determined by the α^2^ and α^1/2^ methods and by the Boltzmann function, can be understood with the help of Fig. 3.

Figure 3 contains a pictorial description of the valence and conduction bands, along with some optical absorption transitions that usually take place in direct and indirect bandgap semiconductors. For simplicity reasons, the transitions presented in Fig. 3 were divided into three main classes: 1– **below bandgap** (low energy) non-absorbing transitions; 2– corresponding to the **absorption onset**, due to the presence of defects (in direct bandgap semiconductors) and/or because of phonon-assisted processes (indirect bandgap); and 3– setting the limits of the **high absorption edge** (nearby ${{\rm{E}}}_{{\rm{0}}}^{{\rm{Boltz}}}$, or the inflection point of the Boltzmann function).

Optical transitions involving photons with even higher energies (beyond transition 3) give rise to practically constant (or very low rate) absorption coefficients. A sketch containing a typical α *versus* E graph (Boltzmann function) is also shown in Fig. 3.

According to this scheme: direct E_gap_’s are defined by optical transitions taking place close to the **high absorption edge** [${{\rm{n}}}_{{\rm{dir}}}^{{\rm{Boltz}}} \sim 0.3$ in Eq. (9)], whereas indirect E_gap_’s are subjected to optical transitions around the **absorption onset** region (${{\rm{n}}}_{{\rm{ind}}}^{{\rm{Boltz}}} \sim 4.3$). Despite the fact that the phenomenological description of Fig. 3 is consistent with the empirical relationship provided by Eq. (9), it is clear that further research is required. Nevertheless, the use of a Boltzmann function to estimate the optical bandgap of semiconductors presents some clear advantages when compared with the traditional α^2^ and α^1/2^ methods. Within them, one can mention: (a) since they will provide the same ${{\rm{E}}}_{{\rm{0}}}^{{\rm{Boltz}}}$ (Fig. 2 and SuppInfo_Part 2), any α(E) formula can be used – some of them do not requiring optical reflection measurements [Eq. (4), for instance]; (b) the E_gap_ values, as determined from Eq. (9), are not susceptible to the fitting range (SuppInfo_Part 3); and (c) the results provided by Eq. (9) are immune to experimental details like optical misalignment and/or inappropriate correction by the response of the optical system (SuppInfo_Part 4). Furthermore, the method based on the Boltzmann function proved to be efficient not only with crystalline semiconductors in the bulk (wafer) form, but also with powder-like samples and amorphous films – as is shown in the following.

### Crystalline (powder) Ge, Si, and GaAs

**Figure 4:**
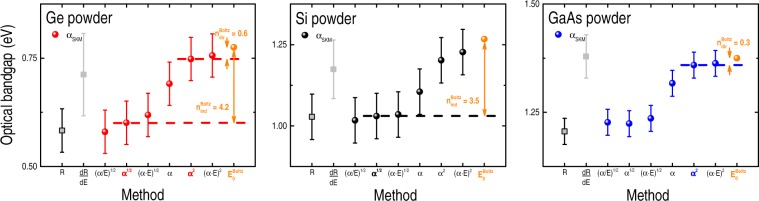
Optical bandgaps of crystalline Ge, Si, and GaAs powder samples – as determined from α_SKM_(E) [Eq. (7)] – according to different experimental approaches: extrapolation (*energy derivative*) of the diffuse reflectance curve R (*dR/dE*); Cody’s bandgap [(α/E)^1/2^ *versus* E plot]; indirect E_gap_ [α^1/2^]; Tauc’s bandgap [(α · E)^1/2^]; extrapolation of α(E); direct E_gap_ [α^2^]; inappropriate version of the Tauc’s bandgap [(α · E)^2^]; and after fitting the α_SKM_(E) spectra with Boltzmann functions (${{\rm{E}}}_{{\rm{0}}}^{{\rm{Boltz}}}$). Error bars correspond to data dispersion due to different sets of measurements–analysis.

The pseudo-absorption function F(R_∞_) [~α_SKM_(E)] of the crystalline Ge, Si, and GaAs powders were achieved from the diffuse optical reflectance measurements by means of Eq. (7) – see SuppInfo_Part 5. The analysis of the α_SKM_(E) spectra gave the optical bandgaps of these materials according to the following experimental approaches: plain extrapolation (and energy derivative) of the diffuse reflectance spectrum; Cody’s and Tauc’s bandgaps (including it’s inappropriate (α · E)^2^ version); and α^2^ and α^1/2^ methods. Likewise, the optical bandgaps of these samples were determined by fitting their respective α_SKM_(E) spectra with Boltzmann functions. The main results are shown in Fig. 4.

Analogous to their crystalline wafer counterparts (Fig. 2), the results of Fig. 4 indicate very similar Cody’s, indirect, and Tauc’s optical bandgaps. It is also evident: higher error bars (~30–70 meV) – mainly due to data dispersion, and ${{\rm{n}}}_{{\rm{dir}}-{\rm{ind}}}^{{\rm{Boltz}}}$ values slightly different from those obtained with the crystalline wafers.

Yet, the optical bandgaps of the powder-like Ge, Si, and GaAs samples can be perfectly described by Eq. (9) with ${{\rm{n}}}_{{\rm{dir}}}^{{\rm{Boltz}}} \sim 0.3$ and ${{\rm{n}}}_{{\rm{ind}}}^{{\rm{Boltz}}} \sim 4.3$, presenting absolute errors (*i.e*., ${{\rm{E}}}_{{\rm{gap}}}^{{\rm{expected}}}-{{\rm{E}}}_{{\rm{gap}}}^{{\rm{Boltz}}}$) below the typical experimental resolution or data dispersion (SuppInfo_Part 1). Even though, one cannot ignore the fact that, because of the crushing-milling process they experienced, the powdered samples do not present the same structural quality of the crystalline wafers.

### Amorphous Ge, Si, and GaAs films

**Figure 5:**
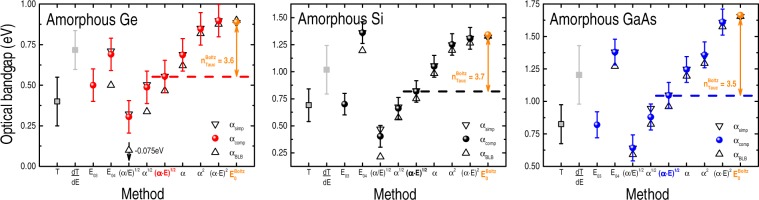
Optical bandgaps of amorphous Ge, Si, and GaAs films – as determined from different α(E) formulas [Eqs (4), (5) and (6)] and different experimental methods: extrapolation (*energy derivative*) of the transmittance curve T (*dT/dE*); Cody’s bandgap [(α/E)^1/2^]; indirect E_gap_ [α^1/2^]; Tauc’s bandgap [(α · E)^1/2^]; extrapolation of α(E); direct E_gap_ [α^2^]; inappropriate version of the Tauc’s bandgap [(α · E)^2^]; and after fitting the α(E) spectra with Boltzmann functions (${{\rm{E}}}_{{\rm{0}}}^{{\rm{Boltz}}}$). The isoabsorption E_03_ and E_04_ bandgaps are also displayed in the figures. Error bars correspond to data dispersion due to different sets of measurements–analysis and, for clarity reasons, were indicated only in the T-, dT/dE-, and α_comp_-related bandgap values.

The optical absorption coefficient of the amorphous Ge, Si, and GaAs films were determined from their transmittance and reflectance spectra by means of Eq. (4) [α_BLB_], Eq. (5) [α_simp_], and Eq. (6) [α_comp_] – see SuppInfo_Part 6. The analysis of α(E) provided the optical bandgaps of the films according to: the plain extrapolation (and energy derivative) of the transmittance spectra; the E_03_ and E_04_ isoabsorption values; the Cody’s and Tauc’s methods; and α^2^ and α^1/2^ approaches (Fig. 5). The E_gap_’s of the films were also determined by fitting their respective α(E) spectra with Boltzmann functions (${{\rm{E}}}_{{\rm{0}}}^{{\rm{Boltz}}}$).

Compared with the previous series of samples, the E_gap_ of the amorphous films exhibited the highest error bars. More precisely, whereas the typical error in the E_gap_ of crystalline wafers stayed below ~20 meV (Fig. 2), and in the 30–70 meV range for the powder-like samples (Fig. 4), it reached 100 meV in the present series of amorphous films (Fig. 5).

These figures contrast with those exhibited by the less conventional procedures to estimate E_gap_ (*i.e*., plain extrapolation or energy derivative of the T, R, or α spectra), in which a considerable error applies, mainly, because of the uncertainties involving the data analysis. In part, the phenomenon is associated with the atomic (structural) and electronic (optical) characteristics of the samples and it will be discussed in the following. The results of Fig. 5 also indicate: (a) a systematic increase of the E_gap_ values, when estimated according to the Cody’s, indirect, Tauc’s, α(E) extrapolation, direct, (α · E)^2^, and Boltzmann approaches; and (b) that ${{\rm{n}}}_{{\rm{amorp}}}^{{\rm{Boltz}}} \sim 3.6$ is the adjustable parameter of Eq. (9) that best describes the optical bandgap of the amorphous films (SuppInfo_Part 1).

Hitherto, the experimental results can be divided into two main groups: relating the crystalline (wafers and powder-like) samples, and the amorphous films. In the former case, direct and indirect optical bandgaps can be obtained by Eq. (9) (with ${{\rm{n}}}_{{\rm{dir}}}^{{\rm{Boltz}}}=0.3$ and ${{\rm{n}}}_{{\rm{ind}}}^{{\rm{Boltz}}}=4.3$), rendering absolute errors in the order of (or below) the associated experimental resolution and/or data dispersion (Figs 2 and 4, and SuppInfo_Part 1). In the case of the crystalline wafers, the E_gap_ values given by the extrapolation of T [*or α(E)*] show great resemblance with those presented by the Cody’s, indirect, and Tauc’s [*or dT/dE*] methods (Fig. 2). Concerning the powder-like samples, the similarity involves the extrapolation of R [*or its energy derivative*] with the Cody’s, indirect, and Tauc’s methods [*or the linear regression of α*^2^] (Fig. 4). Regarding the amorphous films, their optical bandgaps were properly determined by ${{\rm{E}}}_{{\rm{gap}}}^{{\rm{Boltz}}}={{\rm{E}}}_{{\rm{0}}}^{{\rm{Boltz}}}-{{\rm{n}}}_{{\rm{amorp}}}^{{\rm{Boltz}}}\times {\rm{\delta }}E$ with ${{\rm{n}}}_{{\rm{amorp}}}^{{\rm{Boltz}}}=3.6$ (Fig. 5 and SuppInfo_Part 1), and the resemblance between the different experimental approaches involves the extrapolation of T with the Cody’s, indirect, Tauc’s, and E_03_ bandgaps; as well as between the energy derivative of T with E_04_, α(E), and the linear regression of α^2^ (Fig. 5).

In all of the above cases (involving either crystalline, powder-like, or amorphous samples) the corresponding ${{\rm{E}}}_{{\rm{0}}}^{{\rm{Boltz}}}$ values were the only ones well-defined and immune to data processing details (Figs 2, 4 and 5 and SuppInfo_Parts 2, 3 and 4).

### (Dis)Order and the Boltzmann function

Further to the straightforward and accurate determination of E_gap_, the results supplied by the Boltzmann function suggest that they can be used to probe details of the atomic–electronic structure of the present semiconductor materials. Part of these structure details came out, indirectly, via the increased experimental error – in which the inherent disorder of the Ge, Si, and GaAs samples contributed with some data dispersion (Figs 2, 4 and 5). Nevertheless, the effect is more pronounced when the Boltzmann-related δE slopes are associated with the Raman data of the Ge, Si, and GaAs samples.

Raman scattering spectroscopy is a well-known and extensively applied technique to investigate the atomic structure of several (in)organic materials^97^. Like most of the optically-based techniques, Raman spectroscopy is convenient, fast, non-destructive, compatible with high spectral–spatial resolution etc.^53^ and, above all, appropriate to evaluate the fine features of semiconductor materials^98^. Owing to its superior sensitivity, small changes in the position and shape of certain Raman phonon lines indicate that modifications are taking place in the atomic structure of the semiconductor^99,100^. It happens because of phonon confinement effects (as imposed by the advent of structural randomness, for example) causing the relaxation of the selection rules^101^. The phenomenon is so effective that it is common practice to associate the line-width of the transverse optical TO phonon mode (ΔΩ_TO_) with the structural (dis)order present in the sample^60,102–105^.

This is in perfect accord with the Raman spectra of the present Ge, Si, and GaAs samples in which the most prominent phonon lines undergo a gradual broadening and red-shifting as their structures change from crystalline to amorphous (SuppInfo_Part 7). In fact, the experimental results indicate a rather good correspondence between the ΔΩ_TO_ and δE parameters, in a clear reference to the role played by structural (dis)order onto the electronic processes occurring in the optical absorption measurements. Whereas ΔΩ_TO_ is closely related to the local order around the Ge–Ge, Si–Si, or Ga–As atoms bondings^59^, the Boltzmann-related δE slope denotes the energy extent over which most of the optical transitions happen. Hence, to increasingly disordered structures will correspond broader energy ranges in which the optical processes take place – either because of the advent of defects (transition 2 in Fig. 3) and/or due to the mixing of optical processes (transitions 2 and 3 in Fig. 3). The effect can be noticed by means of the energy derivative of the Boltzmann function, making evident the energy range in which the optical absorption rate is more prominent, *i.e*., different from zero or constant (SuppInfo_Part 8). A by-product of such procedure is the achievement of equivalent E_gap_ empirical relationships. In fact, the derivation of any sigmoid function results in a bell-shaped curve^92,93^ that, in the present case is a Gauss function characterized by an average energy ${{\rm{E}}}_{{\rm{0}}}^{{\rm{Gauss}}}$ ($={{\rm{E}}}_{{\rm{0}}}^{{\rm{Boltz}}}$) and standard deviation $\sigma$. On these grounds:

10 $${{\rm{E}}}_{{\rm{gap}}}^{{\rm{Gauss}}}={{\rm{E}}}_{{\rm{0}}}^{{\rm{Gauss}}}-{{\rm{n}}}_{{\rm{type}}}^{{\rm{Gauss}}}\times {\rm{\sigma }},$$

where the adjustable parameter ${{\rm{n}}}_{{\rm{type}}}^{{\rm{Gauss}}}$ assumes the values: ${{\rm{n}}}_{{\rm{dir}}}^{{\rm{Gauss}}}=0.2$, ${{\rm{n}}}_{{\rm{ind}}}^{{\rm{Gauss}}}=2.7$, and ${{\rm{n}}}_{{\rm{amorp}}}^{{\rm{Gauss}}}=2.3$ – still producing E_gap_’s with absolute errors consistent with the experimental resolution and data dispersion. Occasionally, the E_gap_ values can be obtained directly from the energy derivative of the α(E) spectrum. However, much of the α(E) experimental spectrum is frequently irregular and/or noisy, making it very difficult to establish E_gap_ with precision.

## Conclusions

The role played by semiconductor materials in advancing the basics of solid-state physics and towards the achievement of (micro-)electronic devices was reviewed by remembering some of their scientific milestones. The importance of optically-based spectroscopic techniques in the semiconductors S&T was also highlighted by presenting the main ideas behind the energy bandgap E_gap_ of materials. Driven by these facts, the optical absorption spectra α(E), and respective E_gap_’s, of certain semiconductors were investigated in detail. The study included the calculation of α(E) by four different formulas (α_BLB_, α_simp_, α_comp_, and α_SKM_) along with the calculation of E_gap_ according to various approaches (involving the linear regression of α, α^2^, α^1/2^, (α · E)^1/2^ etc.).

A new methodology to determine E_gap_, based on the fit of α(E) with a sigmoid-Boltzmann function, was considered too. To validate the Boltzmann-related method, its performance was contrasted with the E_gap_’s provided by the standard approaches, when applied to the semiconductors Si, Ge, and GaAs – under the bulk, powder, and amorphous (film) forms. In addition to Si, Ge, and GaAs, other semiconductor materials (12 in total) have been considered, rendering an empirical relationship that proved to be very efficient and reliable.

According to these findings, the optical bandgap can be given by ${{\rm{E}}}_{{\rm{gap}}}={{\rm{E}}}_{{\rm{0}}}^{{\rm{Boltz}}}-{{\rm{n}}}_{{\rm{type}}}^{{\rm{Boltz}}}\times {\rm{\delta }}E$, where ${{\rm{E}}}_{{\rm{0}}}^{{\rm{Boltz}}}$ and δE correspond to the central energy and slope of the Boltzmann function; and the adjustable parameter ${{\rm{n}}}_{{\rm{type}}}^{{\rm{Boltz}}}$ stands for the type of optical transition (or bandgap): ${{\rm{n}}}_{{\rm{dir}}}^{{\rm{Boltz}}}=0.3$, ${{\rm{n}}}_{{\rm{ind}}}^{{\rm{Boltz}}}=4.3$, and ${{\rm{n}}}_{{\rm{amorp}}}^{{\rm{Boltz}}}=3.6$.

The whole process is supported by a simple phenomenological model, that is perfectly consistent with the experimental results. Furthermore, compared with the standard approaches to determine E_gap_, the Boltzmann-related method revealed to be immune to measurements–analyses details (including accidental optical misalignment and/or improper spectra correction, α(E) formula, fitting range etc.) and, above all, it was able to provide bandgap values on the order of (or below) the experimental error characteristic of the conventional methods to determine E_gap_. Finally, the Boltzmann-related δE slope is very sensitive to the structural-electronic characteristics of the material and, therefore, it seems appropriate to probe the (dis)order of the semiconductor.

## Materials and Methods

High-quality commercial wafers of crystalline Ge (c-Ge), Si (c-Si), and GaAs (c-GaAs) have been considered in the present work. They correspond to wafers undoped, mirror-polished on both sides, and concerning their orientation and thickness: c-Ge (111) and (250 ± 5) μm, c-Si (100) and (400 ± 5) μm, and c-GaAs (100) and (500 ± 5) μm. The powder version of Ge (pw-Ge), Si (pw-Si), and GaAs (pw-GaAs) were obtained by crushing–milling the previous wafers in a mortar-pestle apparatus made of agate. The procedure resulted in semiconductor particles typically in the 50–200 μm range, as indicated by optical microscopy. The set of amorphous samples comprised films of Ge (a-Ge), Si (a-Si), and GaAs (a-GaAs). The films were deposited onto fused silica substrates by sputtering the corresponding solid targets (high-purity Ge, Si, or GaAs) in an atmosphere of argon gas. On the basis of earlier studies^104,106,107^, the films proved to be undoped and with thicknesses equal to: a-Ge (500 ± 10) nm; a-Si (2000 ± 50) nm; and a-GaAs (1000 ± 50) nm. The crystalline or amorphous character of all samples was verified through Raman scattering spectroscopy (632.8 nm laser excitation). Also, the error in the sample thicknesses took into account the data dispersion due to micrometer screw (wafers) or surface profilometer (films) measurements. Regarding the optical diffuse reflection measurements of the powder-like samples, a 3 mm high amount of material was considered.

The optical transmission–reflection measurements of the Ge, Si, and GaAs samples were carried out along the visible (VIS) and near-infrared (NIR) ranges: from ~500 to 2500 nm (corresponding to the ~2.5–0.5 eV energy interval). The experimental setup comprised miniature optical spectrophotometers (Ocean Optics HR4000 and NIRQuest256-2.5 in the VIS and NIR ranges), optical fibers, fiber-related lenses, and special accessories (bifurcated fibers and integrating sphere for the reflection measurements). The experiments took place at room-temperature by keeping the incident light (VIS–NIR extended source) normal to the surface of the samples. Sample analyzed areas and spectral resolution remained around 3 mm^2^ (2 mm spot diameter) and below 10 nm, respectively. All spectra were properly corrected by the optical response of the system (light source + diffraction grating + detector) to ensure 100% transmission or reflection of light. Moreover, either specular (aluminum mirror) or diffuse (Spectralon®) reflectance standards were considered to measure the crystalline wafers, the amorphous films, and the powder-related samples. Concerning the amorphous films, their spectra were also corrected by the fused silica substrate. The experimental work included the acquisition of various spectra and the measurement of different regions of the very same sample.

## Supplementary information

Supplementary Information
