# Supplementary material for: Revisiting the optical bandgap of semiconductors and the proposal of a unified methodology to its determination
Source: Sci Rep. 2019 Aug 2;9:11225. doi: 10.1038/s41598-019-47670-y (PMC6677798; doi:10.1038/s41598-019-47670-y)
Supplement: Supplementary file 1 — Supplementary Information [file 41598_2019_47670_MOESM1_ESM.pdf]

Supplementary Information

The following material complements the results presented in the article

"*Revisiting the optical bandgap of semiconductors and the proposal of a unified methodology to its determination*"

by A. R. Zanatta (IFSC – USP – Brazil).

SuppInfo_Part1
Table with a summary of the characteristics and optical bandgap of all semiconductors considered in this study;

SuppInfo_Part2
According to the present method (based on the use of a sigmoid-Boltzmann function), any $\alpha(E)$ formula can be used to determine E_{gap};

SuppInfo_Part3
Contrary to the conventional α^2 and $\alpha^{1/2}$ methods, the E_{gap} value as provided by E_0^{Boltz} does not depend on the fitting range;

SuppInfo_Part4
Influence of measurement details (optical misalignment & inappropriate system response correction);

SuppInfo_Part5
Optical spectra – diffuse reflectance, pseudo-absorption coefficient, and α^2 and $\alpha^{1/2}$ plots – related to Ge, Si, and GaAs powders;

SuppInfo_Part6
Optical spectra – transmittance, reflectance, absorption coefficient, and $(\alpha / E)^{1/2}$ and $(\alpha \cdot E)^{1/2}$ plots – related to Ge, Si, and GaAs films;

SuppInfo_Part7
Raman spectra and (dis)order correlation of crystalline (wafer and powder) and amorphous (films) Ge, Si, and GaAs samples;

SuppInfo_Part8
Optical absorption spectra of Ge, Si, and GaAs samples, along with their fitted Boltzmann functions and respective energy derivatives.

SuppInfo_Part1– Table with all the semiconductor materials considered in the present work: 12 in total (09 direct bandgap + 02 indirect + 01 dir/ind). The Table includes information of semiconductors under the crystalline (xtal), powdered (pwd), and amorphous (film) forms and, except for the type of bandgap (direct or indirect) and reference values (E_{gap}^{liter}), all data were achieved after experimental analyses. The Table presents the bandgap values from the conventional direct (α^2), indirect ($\alpha^{1/2}$), and Tauc [$(\alpha \cdot E)^{1/2}$] methods, as well as the data provided from Boltzmann functions, *i.e.*: E_0^{Boltz} and δE. Additionally, the Table shows the absolute error

$$\Delta E = E_{gap} - E_{gap}^{Boltz}, \qquad (1 – SuppInfo\_Part1)$$

where E_{gap} corresponds to the optical bandgaps achieved by the α^2, $\alpha^{1/2}$, and $(\alpha \cdot E)^{1/2}$ approaches; and

$$E_{gap}^{Boltz} = E_0^{Boltz} - n_{type}^{Boltz} \cdot \delta E \qquad (2 – SuppInfo\_Part1)$$

with the n_{type}^{Boltz} averaged values (Fig 1_Part1): $n_{dir}^{Boltz} = 0.3$, $n_{ind}^{Boltz} = 4.3$, and $n_{amorp}^{Boltz} = 3.6$. Accordingly, the absolute errors are consistent with the typical experimental errors (including spectrum resolution and data dispersion), *i.e.*: < 20 meV (crystalline wafers), < 70 meV (powder samples), and < 100 meV (amorphous films).

Table – Summary of the optical bandgap data of all semiconductor materials investigated in this work.

Semicond	Band Gap	E_{gap}^{liter} (eV)	α^2 (eV)	$\alpha^{1/2}$ (eV)	$(\alpha \cdot E)^{1/2}$ (eV)	E_0^{Boltz} (eV)	δE (meV)	n_{type}^{Boltz}	ΔE (meV)
Si (xtal)[1]	ind	1.11	–	1.10(2)	–	1.27(4)	40	4.3	0
Si (pwd)	ind	–	–	1.02(7)	–	1.26(7)	68	3.5	50
Ge (xtal)[1]	dir	0.80	0.76(5)	–	–	0.77(2)	26	0.3	0
Ge (xtal)[1]	ind	0.67	–	0.65(3)	–	0.77(2)	26	4.5	7
Ge (pwd)	dir	–	0.74(8)	–	–	0.77(5)	41	0.6	14
Ge (pwd)	ind	–	–	0.60(1)	–	0.77(5)	41	4.2	2
GaN[1]	dir	3.40	3.34(9)	–	–	3.35(5)	19	0.3	0
GaP[1]	ind	2.25	–	2.21(7)	–	2.30(6)	22	4.0	5
GaAs (xtal)[1]	dir	1.43	1.42(0)	–	–	1.42(3)	9	0.3	0
GaAs (pwd)	dir	–	1.35(9)	–	–	1.37(5)	49	0.3	0
ZnO (xtal)[1]	dir	3.20	3.17(9)	–	–	3.18(8)	21	0.4	3
ZnO (pwd)	dir	–	3.30(8)	–	–	3.34(5)	36	1.0	26
β-ZnS[1]	dir	3.60	3.60(5)	–	–	3.61(4)	35	0.2	1
ZnSe[1]	dir	2.58	2.64(3)	–	–	2.64(8)	12	0.4	1
ZnTe[1]	dir	2.28	2.22(9)	–	–	2.23(3)	11	0.3	0
CdS[1]	dir	2.53	2.38(9)	–	–	2.39(4)	12	0.4	1
CdSe[1]	dir	1.74	1.67(9)	–	–	1.68(3)	9	0.4	1
R-TiO_2 (xtal)[2]	dir	3.0–3.2	3.01(4)	–	–	3.02(3)	22	0.4	2
R-TiO_2 (pwd)	dir	–	3.22(5)	–	–	3.27(0)	50	0.9	30
A-TiO_2 (pwd)	ind	–	–	3.32(1)	–	3.62(3)	86	3.5	66
Si (film)[3]	amorp	0.80	–	–	0.81(8)	1.34(7)	140	3.7	25
Ge (film)[4]	amorp	0.70	–	–	0.55(4)	0.88(9)	93	3.6	0
GaAs (film)[5]	amorp	1.25	–	–	1.04(7)	1.66(5)	175	3.5	12

[1] SuppInfo – Pankove, J. I. in *Optical processes in semiconductors* (Dover Pub., New York, 1971). Appendix II. ISBN 0-486-60275-3

[2] SuppInfo – Hanaor, D. A. H. & Sorrell, C. C. Review of the Anatase to Rutile phase transformation. *J. Mater. Sci.* **46**(4), 855–874 (2011). doi: 10.1007/s10853-010-5113-0

[3] SuppInfo – Zanatta, A. R., Mulato, M. & Chambouleyron, I. Exponential absorption edge and disorder in column IV amorphous semiconductors. *J. Appl. Phys.* **84**(9), 5184–5190 (1998). doi: 10.1063/1.368768

[4] SuppInfo – Zanatta, A. R. & Chambouleyron, I. Nitrogen in the amorphous-germanium network: From high dilution to the alloy phase. *Phys. Rev. B* **48**(7), 4560–4570 (1993). doi: 10.1103/PhysRevB.48.4560

[5] SuppInfo – Zanatta, A. R. 1540 nm light emission from Er-doped amorphous GaAsN films. *Appl. Phys. Lett.* **75**(21), 3279–3281 (1999). doi: 10.1063/1.125324

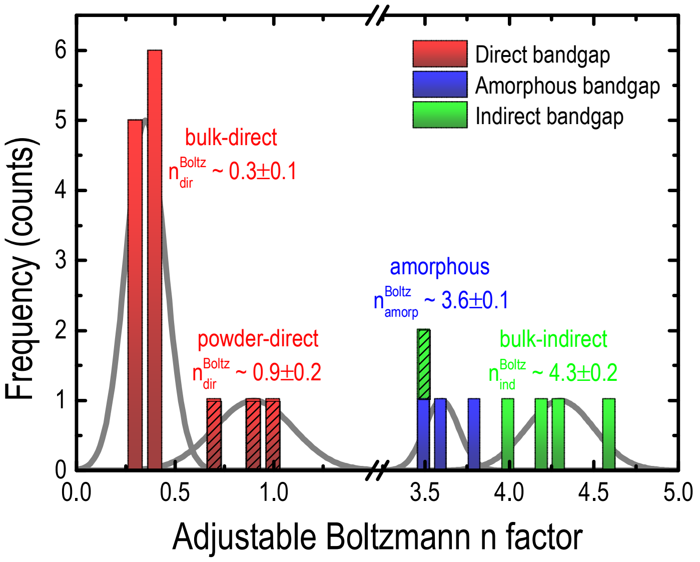

Figure1_Part1– Histogram of the adjustable Boltzmann factors n_{dir}^{Boltz}, n_{ind}^{Boltz}, and n_{amorp}^{Boltz} – corresponding to the data presented in the previous Table. Illustrative Gaussian profiles (centered at n_{type}^{Boltz} and with standard deviations of 0.1 or 0.2) are also shown.

SuppInfo_Part2– Spectra related to the crystalline (wafer) Ge sample. As can be seen, any $\alpha(E)$ formula can be used (some of them do not requiring reflectance measurements – *i.e.*, α_{BLB}) since they will provide the same E_0^{Boltz} and, consequently, E_{gap} value.

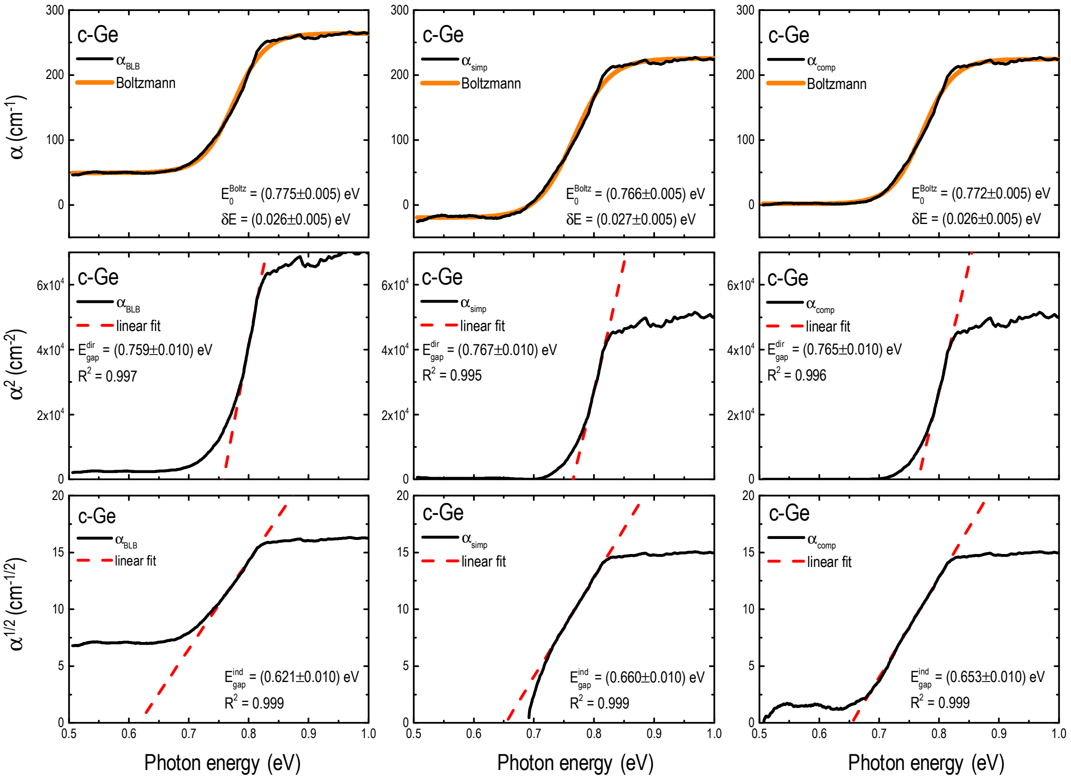

Figure2_Part2– Optical absorption coefficients α_{BLB} [as obtained from Eq. (4)], α_{simp} [Eq. (5)], and α_{comp} [Eq. (6)] of a 250 μm thick crystalline Ge wafer [(111) oriented], along with their corresponding sigmoid-Boltzmann fitted functions. The α^2 and $\alpha^{1/2}$ plots, and respective direct and indirect optical bandgap E_{gap} analyses-values are indicated as well.

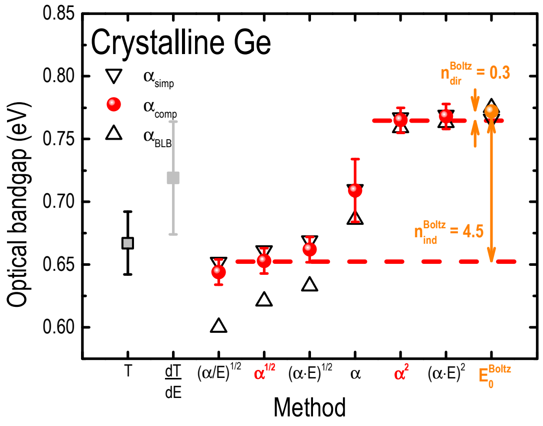

Figure3_Part2– [Reproduction of **Fig. 2** – main text] Optical bandgap E_{gap} (as obtained from α_{BLB}, α_{simp}, and α_{comp}) of a 250 μm thick crystalline Ge wafer [(111) oriented], according to different experimental approaches: extrapolation (*energy derivative*) of the transmittance curve T (*dT/dE*); Cody's bandgap [$(\alpha/E)^{1/2}$ *versus* E plot]; indirect E_{gap} [$\alpha^{1/2}$]; Tauc's bandgap [$(\alpha \cdot E)^{1/2}$]; extrapolation of $\alpha(E)$; direct E_{gap} [α^2]; inappropriate version of the Tauc's bandgap [$(\alpha \cdot E)^2$]; and after fitting the $\alpha(E)$ spectra with a Boltzmann function (E_0^{Boltz}). Error bars correspond to data dispersion due to different sets of measurements–analyses and, for clarity reasons, were indicated only in the T-, dT/dE-, and α_{comp}-related bandgap values. In all cases $E_0^{Boltz} \pm 0.005$ eV, *i.e.*, on the order of (or below) the typical spectrum resolution of ~ 10 nm.

SuppInfo_Part3– Contrary to the E_{gap}'s provided by the conventional α^2 and $\alpha^{1/2}$ methods, the fitting of $\alpha(E)$ with a Boltzmann function yields E_0^{Boltz} values that are not susceptible to the fitting range. In the following analysis, one have considered the four different fitting ranges: LOW (0.70–0.75 eV) **direct** & (0.64–0.68 eV) **indirect**, MIDdle (0.79–0.81 eV) **dir** & (0.70–0.74 eV) **ind**, HIGH (0.81–0.83 eV) **dir** & (0.76–0.81 eV) **ind**, FULL (0.70–0.83 eV) **dir** & (0.64-0.81 eV) **ind**. In the case of the sigmoid-Boltzmann functions (see **Figure2_Part2**), the fitting region was 0.50–1.00 eV.

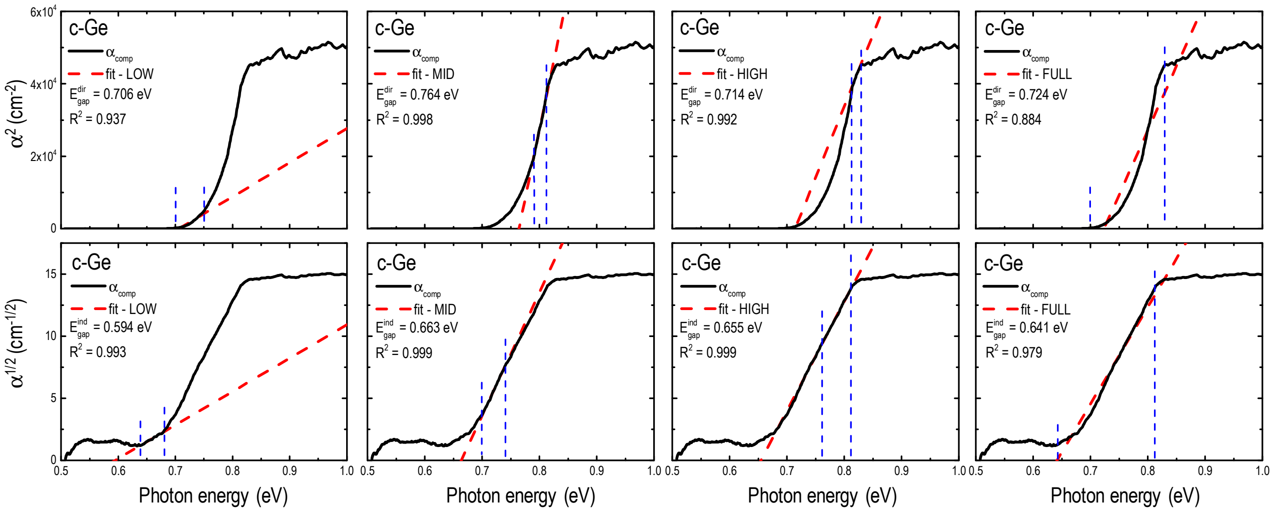

Figure4_Part3– α^2 and $\alpha^{1/2}$ plots of a 250 μm thick crystalline Ge wafer [(111) oriented]. The corresponding direct and indirect E_{gap} values were obtained after the linear regression analysis of α^2 and $\alpha^{1/2}$ at different fitting ranges: LOW, MID, HIGH, and FULL – as indicated in the figures.

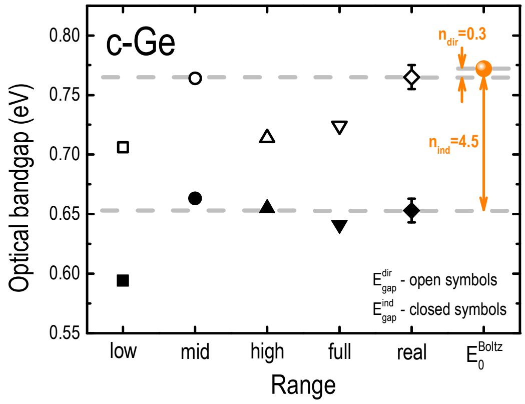

Figure5_Part3– Optical bandgap E_{gap} of a 250 μm thick crystalline Ge wafer [(111) oriented], as obtained from the linear regression analysis of α^2 and $\alpha^{1/2}$ plots by adopting different fitting ranges (see **Figure4_Part3**): <u>low</u> (0.70–0.75 eV) direct & (0.64–0.68 eV) indirect, <u>mid</u> (0.79–0.81 eV) dir & (0.70–0.74 eV) ind, <u>high</u> (0.81–0.83 eV) dir & (0.76–0.81 eV) ind, <u>full</u> (0.70–0.83 eV) dir & (0.64-0.81 eV) ind, and <u>real</u> (as considered in the main text). In the case of the sigmoid-Boltzmann function (E_0^{Boltz}), the fitting region ranged from 0.50 to 1.00 eV and $E_0^{Boltz} \pm 0.005$ eV.

SuppInfo_Part4– Experimental data illustrating the insensitivity of E_0^{Boltz} – and respective E_{gap} values – to measurement problems like optical misalignment and/or inappropriate spectrum correction by the optical response. For comparison purposes, the analysis took into consideration the α_{comp} spectrum (indentified as **real**) along with 10% variations due to: **upward shift** ($\alpha_{comp} + 0.1\,\alpha_{comp}$); **downward shift** ($\alpha_{comp} - 0.1\,\alpha_{comp}$); **stretched** ($1.1 \times \alpha_{comp}$); and **compressed** ($0.9 \times \alpha_{comp}$). In all of these cases the sigmoid-Boltzmann functions yielded exactly the same E_0^{Boltz} (0.772 eV) and δE (0.026 eV) values. The data refer to a 250 μm thick crystalline Ge wafer [(111) oriented].

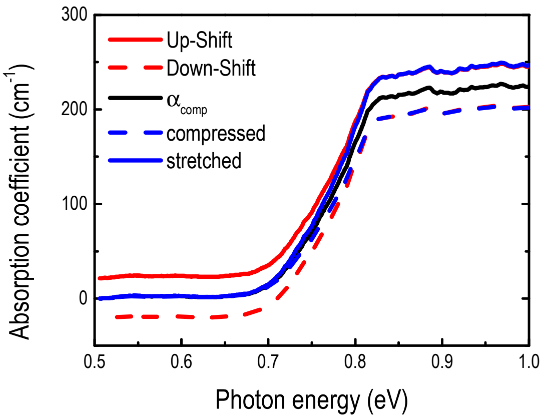

Figure6_Part4– Optical absorption coefficient (α_{comp}) of a crystalline Ge wafer illustrating measurement errors like misalignment (upward and downward vertical shifts) and improper system response correction (compressed and stretched spectra).

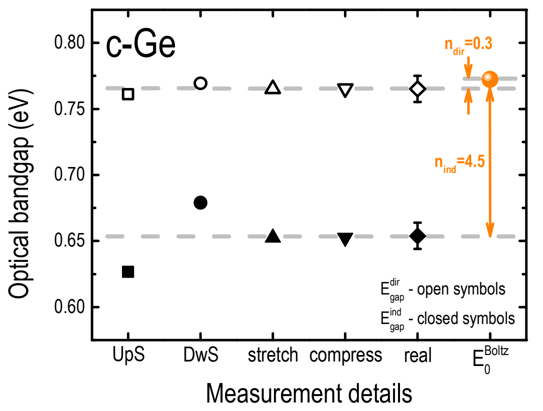

Figure7_Part4– Optical bandgap E_{gap} of a crystalline Ge wafer, as obtained from the linear regression of α^2 and $\alpha^{1/2}$ plots in the very same fitting range: direct bandgap (0.79–0.81 eV) & indirect (0.70–0.74 eV) – always rendering $R^2 > 0.99$. In the case of the sigmoid-Boltzmann functions (E_0^{Boltz}), the fitting range was 0.50–1.00 eV and $E_0^{Boltz} \pm 0.005$ eV.

SuppInfo_Part5– Diffuse optical reflectance, pseudo-absorption function $[F(R_\infty) \sim \alpha_{SKM}(E)]$, and α^2 and $\alpha^{1/2}$ plots of Ge, Si, and GaAs powders.

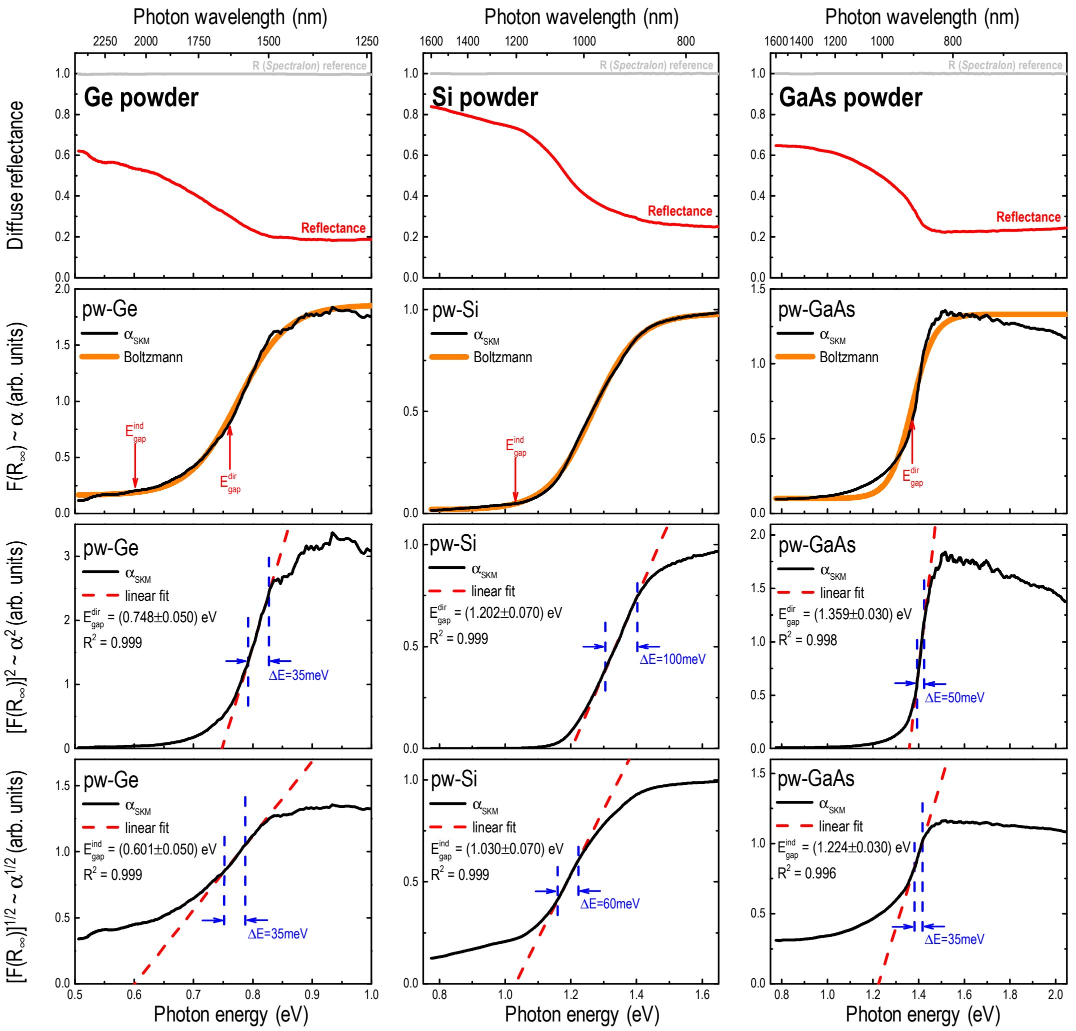

Figure8_Part5– Diffuse optical reflectance, and pseudo-absorption function $F(R_\infty)$ $[\sim \alpha_{SKM}(E)$, after Eq. (7)] spectra of crystalline Ge, Si, and GaAs powder samples. The measurements were carried out at room-temperature and were corrected by system response and diffuse reflection (Spectralon®) standard. Direct and indirect optical bandgaps were obtained from the α^2 and $\alpha^{1/2}$ plots – clearly indicating the resultant linear fit, the fitting range ΔE, and the R^2 *goodness-of-fit* measure. The errors in the E_{gap} values refer to data dispersion due to different measurements runs. The α *versus* E graphs also show the Boltzmann functions used to fit $\alpha_{SKM}(E)$, as well as the E_{gap} values obtained from the α^2 and $\alpha^{1/2}$ plots. In all cases $E_0^{Boltz} \pm 0.005$ eV, *i.e.*, on the order of (or below) the typical spectra resolution of ~ 10 nm.

SuppInfo_Part6– Optical transmittance, reflectance, and absorption coefficient of amorphous Ge, Si, and GaAs films deposited onto fused silica substrates.

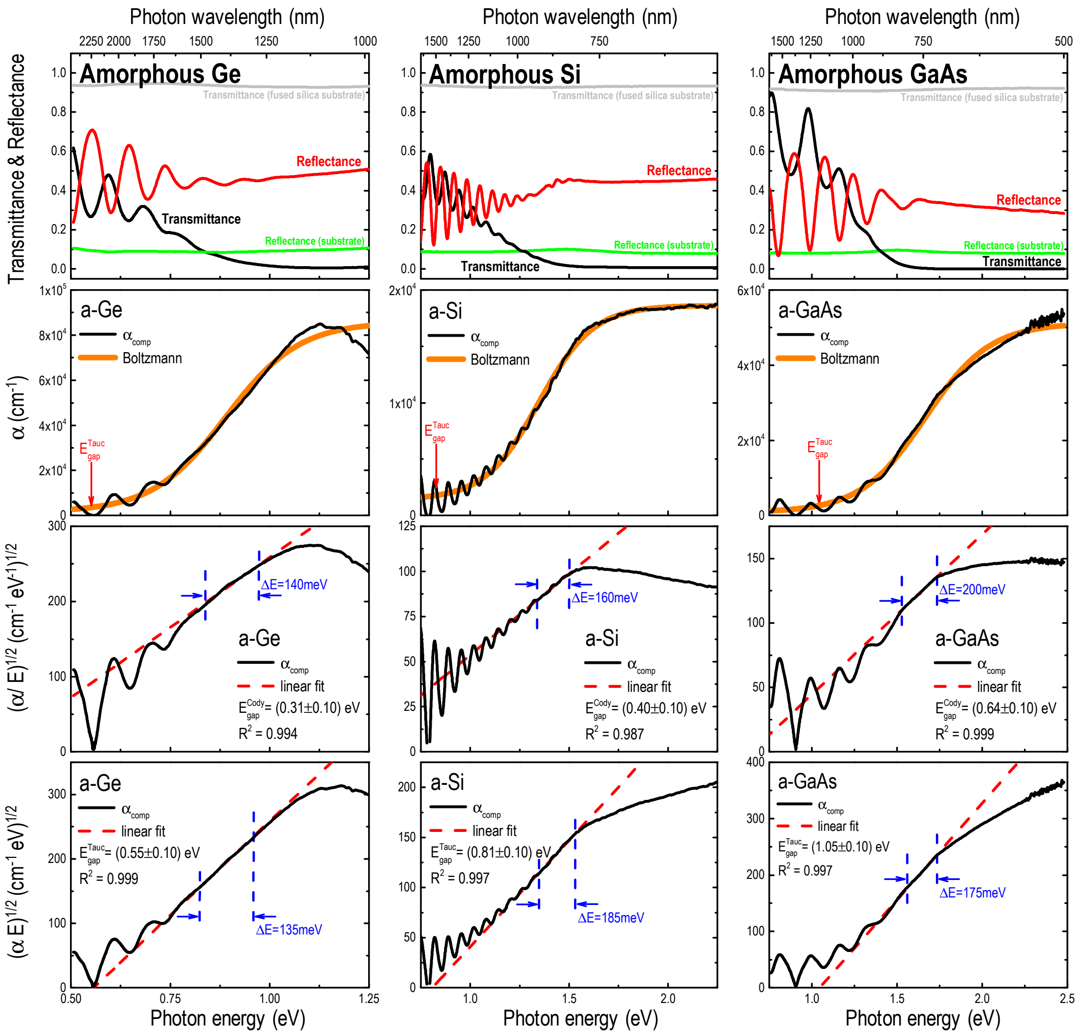

Figure9_Part6– Optical transmittance, reflectance, and absorption coefficient $\alpha(E)$ [after Eq. (6)] spectra of amorphous Ge, Si, and GaAs films. The measurements were carried out at room-temperature and were corrected by system response, specular reflection (Al mirror) standard, and fused silica substrate. Both Cody's and Tauc's optical bandgaps were determined from their respective $(\alpha / E)^{1/2}$ and $(\alpha \cdot E)^{1/2}$ graphic representations – clearly indicating the resultant linear fit, fitting range ΔE, and R^2 *goodness-of-fit* measure. The error in the E_{gap} values refer to data dispersion due to different measurements runs. The α *versus* E graphs also show: the Boltzmann functions used to fit $\alpha(E)$ and the Tauc's E_{gap} values. In all cases $E_0^{Boltz} \pm 0.005$ eV, *i.e.*, on the order of (or below) the typical spectra resolution of ~ 10 nm. The fringes apparent in all spectra originated from light interference effects at the air–film–substrate interfaces.

SuppInfo_Part7– Raman spectra of the Ge, Si, and GaAs samples, together with the identification of their principal phonon modes: transverse acoustical (TA), transverse (TO) and longitudinal optical (LO), and overtones[6-8]. According to the figure, the most prominent phonon lines undergo a gradual broadening and red-shifting as the samples change from crystalline to amorphous. In fact, when the atomic structure is completely disordered, the Raman spectrum of the amorphous film can be qualitatively interpreted as a blurred version of the vibrational density of states of its crystalline counterpart.

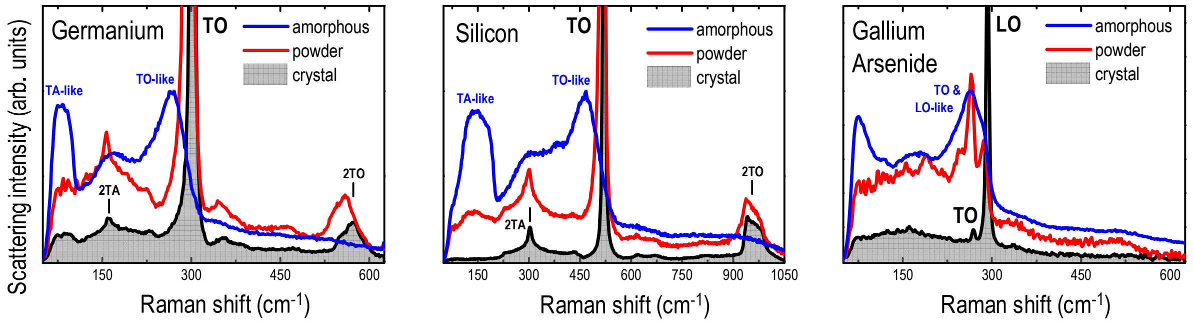

Figure10_Part7– Raman scattering spectra of Ge, Si, and GaAs samples under the crystal (wafer), powder, and amorphous (film) forms. The (nonpolarized) Raman spectra were obtained at room-temperature with 638.2 nm photon excitation (laser cutoff at 70 cm^{-1}). The main phonon modes [transverse acoustical (TA), transverse (TO) and longitudinal optical (LO), and overtones] are indicated in the figures.

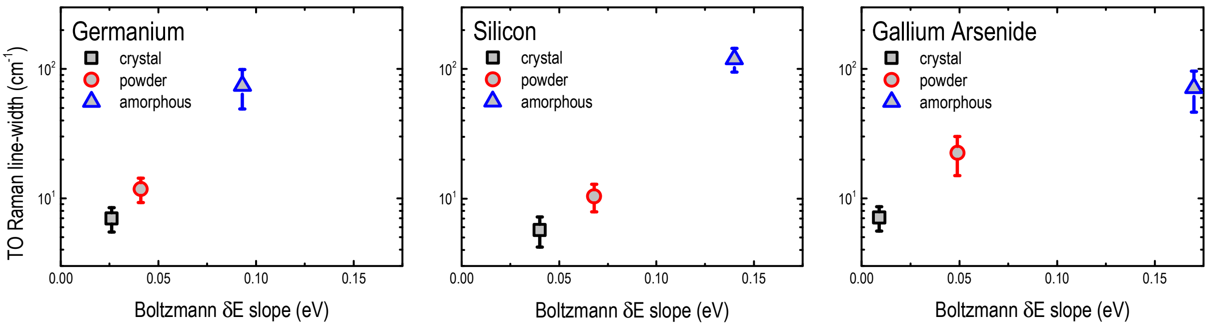

Figure11_Part7– Transverse optical TO Raman line-width $\Delta\Omega_{TO}$ as a function of the Boltzmann δE slope indicating the increased disorder as the atomic structure of the Ge, Si, and GaAs semiconductors change from crystalline (perfectly ordered) to amorphous (disordered).

[6] SuppInfo – Parker Jr, J. H., Feldman, D. W. & Ashkin, M. Raman scattering by silicon and germanium. *Phys. Rev.* **155**(3), 712–714 (1967). doi: 10.1103/PhysRev.155.712

[7] SuppInfo – Smith Jr, J. E., Brodsky, M. H., Crowder, B. L., Nathan, M. I. & Pinczuk, A. Raman spectra of amorphous Si and related tetrahedrally bonded semiconductors. *Phys. Rev. Lett.* **26**(11), 642–646 (1971). doi: 10.1103/PhysRevLett.26.642

[8] SuppInfo – Brafman, O. & Manor, R. Raman line asymmetry in alloys and in ion-implanted polar crystals. *Phys. Rev. B* **51**(11), 6940–6946 (1995). doi: 10.1103/PhysRevB.51.6940

SuppInfo_Part8– Graphs of Ge, Si, and GaAs samples illustrating their optical absorption coefficient spectra [Eq. (6)], fitted Boltzmann functions and respective energy derivatives.

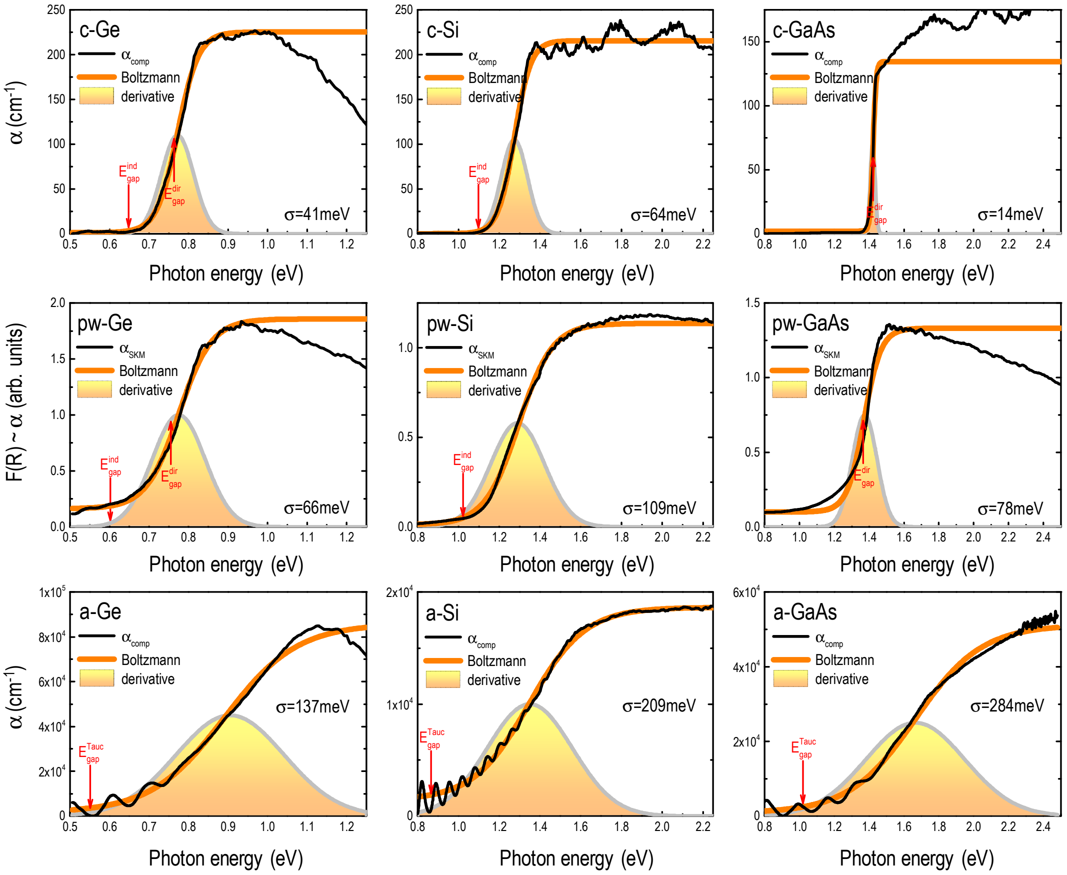

Figure12_Part8– Optical absorption coefficient α_{comp} [Eq. (6)] spectra, Boltzmann functions and respective energy derivatives of Ge, Si, and GaAs samples. The optical bandgap values, as determined from the traditional α^2, $\alpha^{1/2}$, and $(\alpha \cdot E)^{1/2}$ methods, are indicated as well. The σ values correspond to the standard deviations of the Gauss functions (derivatives of the Boltzmann curves).
